# Maternal adverse childhood experiences and health-related quality of life in preschool children: a cross-sectional study

**DOI:** 10.1186/s13034-023-00570-6

**Published:** 2023-02-06

**Authors:** Dezhong Chen, Li Lin, Chunrong Li, Weiqing Chen, Yuying Zhang, Yan Ren, Vivian Yawei Guo

**Affiliations:** 1 Department of Epidemiology, School of Public Health, Sun Yat-Sen University, 74 Zhongshan Second Road, Guangzhou, 510080 Guangdong China; 2 Chengdu Women’s and Children’s Central Hospital, School of Medicine, University of Electronic Science and Technology of China, Chengdu, China; 3 Department of Child Healthcare, Shenzhen Longhua Maternity and Child Healthcare Hospital, Shenzhen, China

**Keywords:** Intergeneration, Adverse childhood experiences, Health-related quality of life, Preschool children

## Abstract

**Background:**

The intergenerational association between maternal adverse childhood experiences (ACEs) and their children’s health-related quality of life (HRQOL) is underexplored. This study aimed to examine such association in Chinese preschool children and to test the moderation role of children’s sex.

**Methods:**

A cross-sectional study was conducted among 4243 mother–child dyads who attended randomly selected preschools. Mothers self-reported their experience of 12 forms of ACEs, including emotional abuse, physical abuse, emotional neglect, physical neglect, intimate partner violence, substance abuse in the household, incarcerated household member, mental illness in household, parental death, parental separation or divorce, bullying, and community violence. Children’s HRQOL was evaluated through mother report of the Pediatric Quality of Life Inventory version 4.0. Linear regression models were established to estimate the associations between maternal ACEs and their children’s HRQOL sub-scores and total scores. Stratified analysis and test for interaction were further conducted to evaluate whether the associations were moderated by children’s sex.

**Results:**

Of the included mothers, 85.8% (n = 3641) had reported exposure to at least one ACE, and 22.3% (n = 948) were exposed to three or more ACEs. Compared to children of mothers without any ACE exposure, those of mothers with 1, 2, or ≥ 3 ACEs all had significantly lower scores of physical, social, and school functioning, as well as lower psychosocial health summary score and total scale score in both crude and adjusted models. However, only children of mothers with two or more ACEs had significantly poorer emotional functioning when compared to their counterparts whose mothers had no ACE exposure. A significant dose-response pattern was also observed between the number of maternal ACEs and children’s HRQOL sub-scores and total scores. Stratified analysis revealed sex-specific pattern between maternal ACEs and their children’s HRQOL. Nonetheless, children’s sex was not a significant moderator.

**Conclusions:**

Our study showed that preschool children of mothers who had any experience of ACEs were at risk of poorer HRQOL. Our findings indicated that screening maternal ACEs in young children and promoting targeted interventions might be a feasible way to mitigate or stop the potential negative intergenerational health and wellbeing implications of ACEs.

**Supplementary Information:**

The online version contains supplementary material available at 10.1186/s13034-023-00570-6.

## Background

Adverse childhood experiences (ACEs) refer to a wide range of potentially stressful or traumatic events that an individual has experienced before the age of 18 years [1], including forms of abuse, neglect, and household dysfunction [2]. Since the landmark study published by Felitti et al. two decades ago [1], cumulative evidence has confirmed a strong, dose-response association of ACEs with a wide range of adverse behavioral and health outcomes in both adults and children [3–10]. A study conducted in the United States has shown that compared to preschool children without exposure to any ACE, their counterparts with experience of three or more ACEs had increased risk of poor teacher-reported academic and behavioral outcomes, such as below-average language and literacy skills, attention problems, social problems, and aggressive behavior [3]. Similar findings were also reported regarding the impact of ACEs experienced in infancy and toddlerhood on poor academic status and maladaptive behaviors at the age of 11 years [4]. Furthermore, a longitudinal study has shown that compared to children with no ACE exposure before the age of three years, the odds of obesity [odds ratio (OR) 2.65, 95% confidence interval (CI) 1.51–4.67,* p*-value < 0.001], respiratory problems (OR 3.18, 95% CI 1.87–5.39,* p*-value < 0.001), and poor health ratings (OR 2.21, 95% CI 1.16–4.21,* p*-value < 0.01) in middle childhood was significantly higher in children with experience of four or more ACEs [5]. A meta-analysis including five longitudinal studies has also demonstrated a positive association between cumulative ACE scores and childhood overweight [6].

Recent years, several studies have further shown that parental exposure to ACEs might affect offspring’s development and health [11–16]. For example, a retrospective cohort study conducted in the United States has shown that both maternal and paternal ACEs were associated with increased risk of suspected developmental delay in their offspring at 2 years of age [11]. A systematic review has further revealed that maternal ACEs were associated with externalizing problems in their offspring across all included studies (n = 11), while the findings were mixed for children’s internalizing problems, with 72.7% (8 out of 11) of the included studies showed a significant association [12]. In addition, there is still debate going on about the gender differences in the association between maternal ACEs and children’s outcomes [13, 15]. A study in Chinese preschool children has found that girls were more likely to have conduct problems than boys when their mothers reported exposure to emotional abuse [13]. Also, girls were more likely to report anxiety compared to boys when their mothers had experience of physical abuse or community violence [13]. In contrast, a study that followed children from birth to two years of age has shown that boys might be more vulnerable than girls when exposed to maternal ACEs [15]. The inconsistent sex-specific findings across aforementioned studies might be caused by the different types of behavioral problems examined, rather than the children’s biological sex. Therefore, there is still a need for further investigations of the intergenerational impact of maternal ACEs on their offspring, as well as the possible moderation role of children’s sex.

Health-related quality of life (HRQOL) is a multidimensional construct that could reflect an individual’s physical, psychological, and social well-being [17]. Reductions in HRQOL scale scores have been linked to cognitive impairment, cardiovascular disease, and even mortality [18–20]. Therefore, measurement of HRQOL has been widely recommended in both clinical practice and research investigations for monitoring overall health [17]. Although previous research has shown that ACE exposure was linked to poorer HRQOL [21], little is known about the intergenerational associations of maternal ACEs with their children’s HRQOL. Mothers with ACE exposure were at higher risk of poorer health, which might lead to their children’s increased ACE exposure [22]. The vicious cycle of ACEs could further cause behavioral problems and poor physical health outcomes in early childhood [23], subsequently contributing to impaired HRQOL in children. Furthermore, maternal exposure to ACEs was associated with problematic parenting practices [24], a key factor that could affect children’s HRQOL [25–27]. Based on aforementioned evidence, we aimed to examine the associations between maternal ACEs and HRQOL in their preschool children in the current study. Stratified analysis and test for interaction were further conducted to evaluate such associations by children’s sex.

## Methods

### Participants

**Fig. 1:**
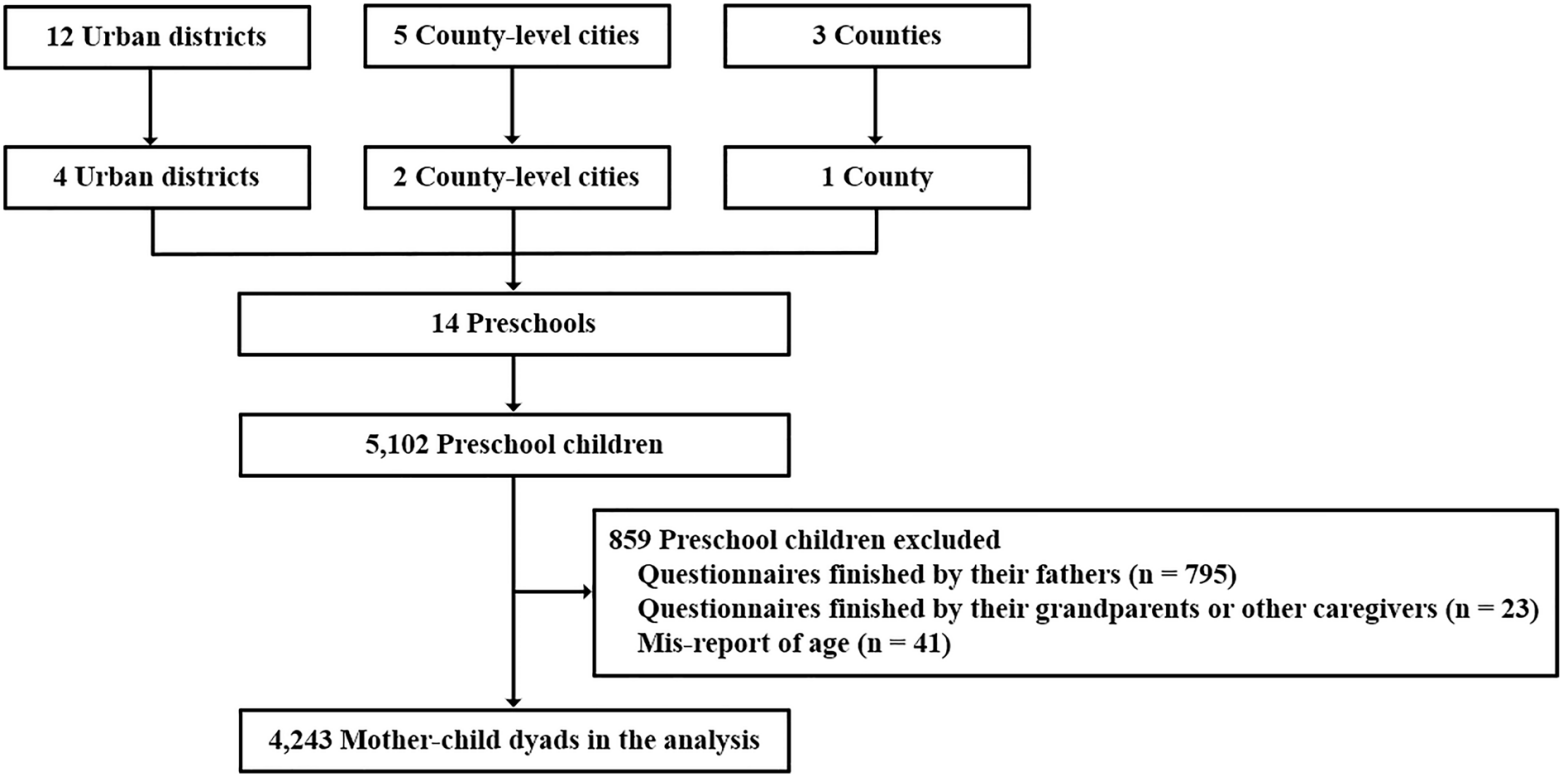
Flow chart of the analytic sample selection process

This cross-sectional study was conducted in Chengdu, a megacity with 12 urban districts, 5 county-level cities, and 3 counties in western China [28]. These three administrative levels were categorized based on factors of urbanization level, industrialization level, and fiscal strength, and so on [29]. To select eligible preschool children, we first randomly selected 4 urban districts, 2 county-level cities, and 1 county. Then, 2 preschools from each selected area were randomly chosen. At last, all children and their parents in these 14 preschools were invited to take part in our study. From May to July 2021, caregivers of 5102 preschool children agreed to join the study and finished an online questionnaire (response rate: 86.5%). We excluded 795 children whose questionnaires were completed by their fathers, 23 children with answers reported by their grandparents or other caregivers, and 41 children with mis-report of age. A total of 4243 mother–child dyads were included in the analysis (Fig. 1).

### Maternal ACEs

Maternal ACEs were measured by the Adverse Childhood Experiences-International Questionnaire (ACE-IQ), a widely used instrument developed by the World Health Organization [30]. It showed good validity and reliability in Chinese population [31]. The questionnaire is comprised of 29 items covering childhood adversities of abuse, neglect, household challenges, and exposure to community and collective violence. Given the sensitivity of sexual abuse in Chinese culture, four relevant items were excluded from the survey. In addition, four items about collective violence were also excluded as this form of adversity was not common in China. The final questionnaire included 21 items covering 12 categories of ACEs, i.e., emotional abuse, physical abuse, emotional neglect, physical neglect, intimate partner violence, substance abuse in the household, incarcerated household member, mental illness in household, parental death, parental separation or divorce, bullying, and community violence. The detailed questionnaire items and definitions of each ACE indicator were listed in Additional file 1: Table S1. For ACE indicator that had multiple questions, a mother was considered as having exposed to this particular form of ACE if she reported a positive answer to any of the related questions. Response to each ACE indicator were dichotomized into yes (coded as 1) or no (coded as 0). Cumulative ACE scores were calculated by summing the 12 ACE indicators for each mother, with a range from 0 to 12. All mothers were further categorized into four groups based on the cumulative ACE score, i.e., 0, 1, 2, and ≥ 3 ACEs.

### Children’s HRQOL

Children’s HRQOL was reported by their mothers using the Pediatric Quality of Life Inventory version 4.0 (PedsQL 4.0) [32], a reliable questionnaire that has been validated with Chinese children [33]. The questionnaire had two versions for children: 1) 2–4 years (21 items), and 2) 5–7 years (23 items). Both versions are comprised of four dimensions, including physical (8 items), emotional (5 items), social (5 items), and school functioning (3 items for 2–4 years old children and 5 items for 5–7 years old children). Each item used a 5-point Likert scale (0 = never a problem, 1 = almost never a problem, 2 = sometimes a problem, 3 = often a problem, and 4 = almost always a problem). All items were first reverse scored and linearly transformed into a 0–100 range. Then, the scores of each dimension were calculated by averaging all items in the corresponding dimension. A psychosocial health summary score was further calculated as the mean value of scores in the emotional, social, and school functioning dimensions. A total scale score was also calculated by averaging scores of all items in the questionnaire. Scores of each dimension and the summary scales ranged from 0 to 100, with higher scores indicating better HRQOL.

### Covariates

#### Children

Children’s age, sex, status of single child, and primary caregivers were reported by their mothers. The status of single child was classified as yes (1 = single child in the family) or no (0 = more than one child in the family). The primary caregivers of children were categorized into 3 groups as mothers (coded as 1), fathers (coded as 2), and grandparents or other people (coded as 3).

#### Mothers

Mothers self-reported their information on age, marital status, educational background, monthly per-capita income, level of family harmony, and negative emotional states. Marital status was dichotomized as married (coded as 1) *versus* unmarried (coded as 2). The latter included single, separated, divorced, and widowed. Monthly per-capita income was categorized into four groups as ≤ 5000 RMB (coded as 1), 5001–10000 RMB (coded as 2), > 10000 RMB (coded as 3), and uncertain (coded as 4), where 1 US $ ≈ 6.96 RMB. Maternal educational background was grouped into three levels as: 1 = junior high school or below, 2 = senior high school, and 3 = bachelor degree or above. The level of family harmony was assessed by the Chinese Family Harmony Scale-5 (FHS-5) [34]. It contained 5 items regarding effective communication, conflict resolution, forbearance, family identity, and quality time within the family. Each item ranged from “do not agree at all” (score 1) to “strongly agree” (score 5). A total score was calculated by summing the 5 items, with higher scores representing greater family harmony. Maternal negative emotional states were evaluated by Depression Anxiety Stress Scales-21 (DASS-21) [35], a self-reported questionnaire that has been demonstrated to be valid and reliable in Chinese adults [36]. The 21-item questionnaire was designed to measure three types of negative emotions, including depression, anxiety, and stress. Each subscale had 7 items that were rated based on a 4-point Likert scale ranging from 0 (did not apply to me at all) to 3 (applied to me very much, or most of the time). Subscale items were summed with higher scores indicating more negative emotions. Cut-off values of 9, 7, and 14 were used to define negative emotional states of depression, anxiety, and stress, respectively.

### Statistical analysis

Descriptive statistics were displayed in mean ± standard deviation (SD) for continuous data and frequency (percentage) for categorical data. Characteristics of both children and mothers across different maternal ACE groups were compared by one-way ANOVA test for continuous variables, and χ^2^ test for categorical variables. To assess the trends in characteristics across the four ACE groups, polynomial comparisons were used to analyze variance in trends for continuous data and the Mantel–Haenszel statistic was used for categorical data. Linear regression models were established to examine the association of maternal ACEs with children’s HRQOL. Model 1 was a crude model. Model 2 adjusted for children’s age, sex, single child status, and primary caregivers, as well as maternal age, marital status, educational background, monthly per-capita income, level of family harmony, and negative emotional states. Dose-response associations between maternal ACEs and children’s HRQOL were further assessed with trend tests. Linearity, normality, homoscedasticity, and absence of multicollinearity were examined for all linear regression models.

Stratified analysis and test for interaction were conducted by children’s sex. Since mothers with negative emotional states might underreport the HRQOL of their children according to the depression-distortion hypothesis [37], we further evaluated the associations between maternal ACEs and children’s HRQOL among mothers without negative emotional states in the sensitivity analysis.

In order to account for statistical power, power analyses were performed to examine the likelihood of multivariate linear regression models detecting specific effect sizes in the current study. The results showed that with an α level of 0.05, the power was high with current sample size (power > 0.98 for HRQOL sub-scores and total scores). Therefore, there was sufficient power to detect small to large effect sizes in the current study.

All data analyses were performed with Stata/SE 15.1 (Stata-Corp, College Station, TX, USA). Statistical significance was two-sided with a *p*-value < 0.05.

## Results

**Fig. 2:**
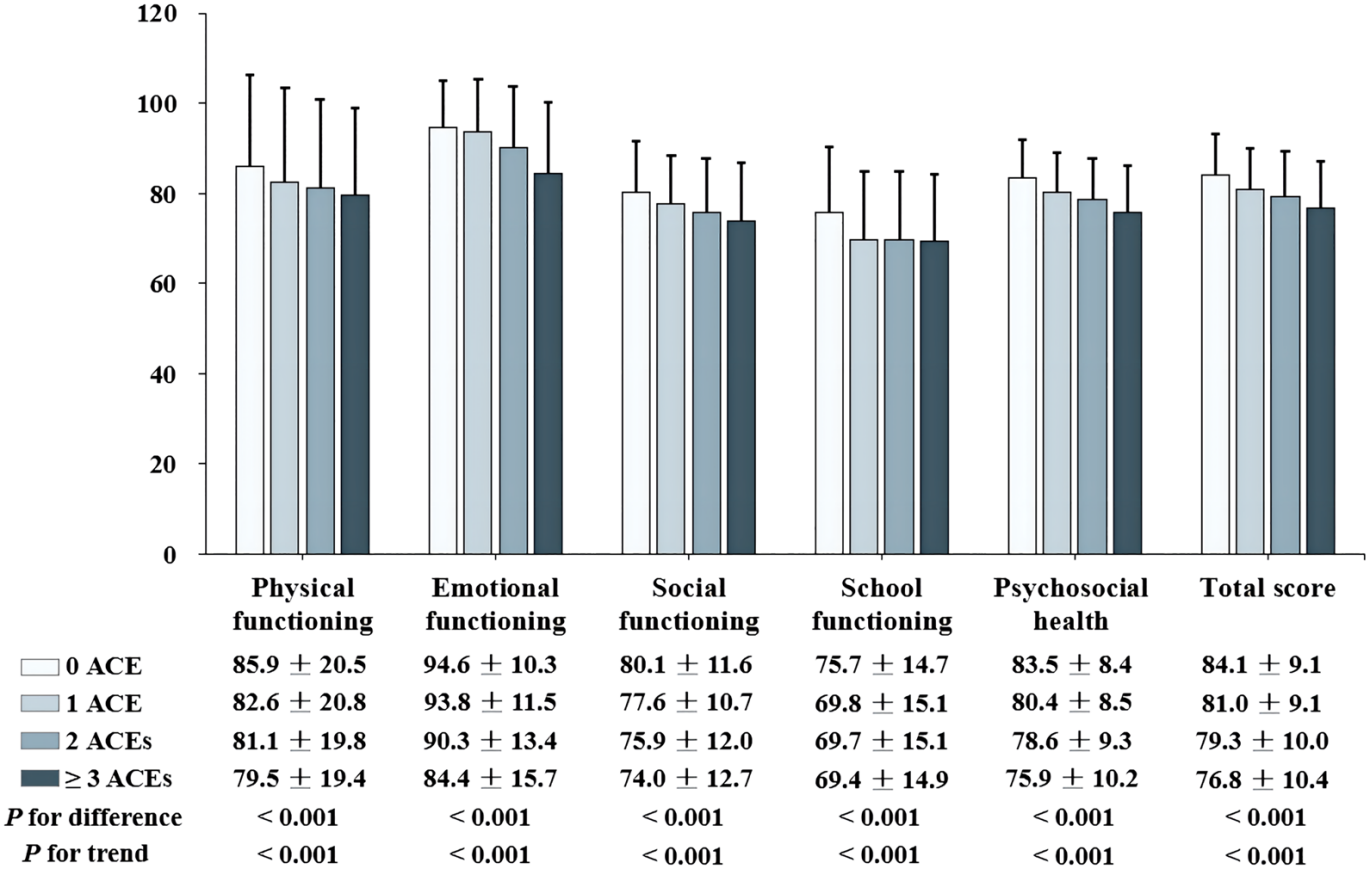
Comparison of children’s HRQOL by the number of maternal ACEs. HRQOL, health-related quality of life; ACEs, adverse childhood experiences

Of the 4243 included mothers, 3641 (85.8%) had experience of at least one ACE, and 948 mothers (22.3%) were exposed to three or more ACEs. The prevalence of included ACE indicators ranged from 0.2% (n = 9 for both household substance abuse and incarcerated household member) to 78.1% (n = 3312 for emotional neglect) (Additional file 1: Table S1). Compared to mothers without any experience of ACEs, greater prevalence of mothers reported unmarried status, low educational background, and negative emotional states in the group with three or more ACEs (Table 1). The mean age of the 4243 children was 4.6 (SD = 1.0) years and approximately half of them were boys (51.7%, n = 2193). Children of mothers who reported three or more ACEs had higher prevalence of being a single child and having a main caregiver of grandparents or other people, compared to those of mothers with no experience of ACEs. The comparison of characteristics by children’s sex was further shown in Additional file 1: Table S2. Except a higher prevalence of maternal depression and better school functioning in girls than boys, no significant difference was observed in other characteristics between different sex. Furthermore, with increasing number of maternal ACEs, we observed significantly lower HRQOL sub-scores and total scores in children (Fig. 2).

**Table 1:** Comparison of characteristics of children and mothers by the number of maternal ACEs

Characteristics	Number of maternal ACEs (n = 4243)				*P*-value for difference	*P*-value for trend
	0 (n = 602)	1 (n = 1919)	2 (n = 774)	≥ 3 (n = 948)		
Maternal characteristics						
Age (years), mean ± SD	33.7 ± 4.6	33.0 ± 4.5	32.9 ± 4.6	33.2 ± 4.7	0.009	0.381
Marital status, n (%)						
Married	588 (97.7%)	1,855 (96.7%)	750 (96.9%)	897 (94.6%)	0.006	0.002
Unmarried	14 (2.3%)	64 (3.3%)	24 (3.1%)	51 (5.4%)		
Educational background, n (%)						
Junior high school or below	23 (3.8%)	205 (10.7%)	80 (10.3%)	79 (8.3%)	< 0.001	0.181
Senior high school	108 (17.9%)	485 (25.3%)	197 (25.5%)	198 (20.9%)		
Bachelor degree or above	471 (78.2%)	1,226 (64.0%)	497 (64.2%)	670 (70.7%)		
Monthly per-capita income, n (%)						
≤ 5000 RMB	135 (22.4%)	569 (29.7%)	209 (27.0%)	262 (27.6%)	< 0.001	0.536
5001–10000 RMB	170 (28.2%)	492 (25.6%)	200 (25.8%)	244 (25.7%)		
> 10000 RMB	240 (39.9%)	587 (30.6%)	261 (33.7%)	341 (36.0%)		
Uncertain	57 (9.5%)	271 (14.1%)	104 (13.4%)	101 (10.7%)		
Negative emotional states, n (%)						
Depression	3 (0.5%)	59 (3.1%)	35 (4.5%)	121 (12.8%)	< 0.001	< 0.001
Anxiety	7 (1.2%)	91 (4.7%)	44 (5.7%)	152 (16.0%)	< 0.001	< 0.001
Stress	6 (1.0%)	64 (3.3%)	39 (5.0%)	135 (14.2%)	< 0.001	< 0.001
Level of family harmony, mean ± SD	22.9 ± 3.6	21.1 ± 4.8	20.5 ± 4.5	19.6 ± 4.4	< 0.001	< 0.001
Child characteristics						
Girls, n (%)	282 (46.8%)	907 (47.3%)	389 (50.3%)	472 (49.8%)	0.339	0.108
Age (years), mean ± SD	4.7 ± 1.0	4.6 ± 1.0	4.6 ± 1.0	4.6 ± 1.0	0.264	0.383
Status of single child, n (%)						
Yes	272 (45.2%)	837 (43.6%)	355 (45.9%)	493 (52.0%)	0.001	< 0.001
No	330 (54.8%)	1,082 (56.4%)	419 (54.1%)	455 (48.0%)		
Primary caregivers, n (%)						
Mothers	470 (78.1%)	1,426 (74.3%)	571 (73.8%)	670 (70.7%)	0.048	0.002
Fathers	18 (3.0%)	69 (3.6%)	23 (3.0%)	37 (3.9%)		
Grandparents or other people	114 (18.9%)	424 (22.1%)	180 (23.3%)	241 (25.4%)		

*ACEs* adverse childhood experiences, *SD* standard deviation

The associations between maternal ACEs and HRQOL in children were shown in Table 2. In Model 1 without any adjustment, we found that compared to children of mothers without any exposure to ACEs, those of mothers with experience of one or more ACEs during childhood had significantly lower scores of physical functioning (β = −3.22, 95% CI −5.11 to −1.34, *p*-value < 0.05 for one ACE; β = −4.72, 95% CI −6.88 to −2.57, *p*-value < 0.05 for two ACEs; and β = −6.41, 95% CI −8.46 to −4.36, *p*-value < 0.05 for ≥ 3 ACEs), social functioning (β = −2.48, 95% CI −3.52 to −1.44, *p*-value < 0.05 for one ACE; β = −4.22, 95% CI −5.47 to −2.97, *p*-value < 0.05 for two ACEs; and β = −6.13, 95% CI −7.36 to −4.90, *p*-value < 0.05 for ≥ 3 ACEs), and school functioning (β = −5.88, 95% CI −7.23 to −4.53, *p*-value < 0.05 for one ACE; β = −5.91, 95% CI −7.49 to −4.32, *p*-value < 0.05 for two ACEs; and β = −6.26, 95% CI −7.77 to −4.75, *p*-value < 0.05 for ≥ 3 ACEs), as well as lower psychosocial health summary score (β = −3.07, 95% CI −3.85 to −2.30, *p*-value < 0.05 for one ACE; β = −4.83, 95% CI −5.77 to −3.88, *p*-value < 0.05 for two ACEs; and β = −7.55, 95% CI −8.48 to −6.61, *p*-value < 0.05 for ≥ 3 ACEs), and total scale score (β = −3.11, 95% CI −3.94 to −2.28, *p*-value < 0.05 for one ACE; β = −4.80, 95% CI −5.81 to −3.79, *p*-value < 0.05 for two ACEs; and β = −7.26, 95% CI −8.24 to −6.28, *p*-value < 0.05 for ≥ 3 ACEs). In contrast, only children of mothers who experienced two or more ACEs showed a statistically significant impairment in their emotional functioning (β = −4.35, 95% CI −5.61 to −3.09, *p*-value < 0.05 for two ACEs; and β = −10.25, 95% CI −11.54 to −8.95, *p*-value < 0.05 for ≥ 3 ACEs). We also observed significant dose-response associations of maternal cumulative ACE scores with children’s HRQOL sub-scores and total scores (all *p*-values < 0.001). The results were consistent in adjusted Model 2, although the risk estimates were attenuated. Similar findings were also reported in sensitivity analysis after excluding mothers with negative emotional states (Additional file 1: Table S3).

**Table 2:** Association between the number of maternal ACEs and children’s HRQOL

	β (95% CI) by number of maternal ACEs				*P*-value for trend
	0	1	2	≥ 3	
Model 1					
Physical functioning	Ref	−3.22 (−5.11, −1.34) *	−4.72 (−6.88, −2.57) *	−6.41 (−8.46, −4.36) *	< 0.001
Emotional functioning	Ref	−0.86 (−1.83, 0.11)	−4.35 (−5.61, −3.09) *	−10.25 (−11.54, −8.95) *	< 0.001
Social functioning	Ref	−2.48 (−3.52, −1.44) *	−4.22 (−5.47, −2.97) *	−6.13 (−7.36, −4.90) *	< 0.001
School functioning	Ref	−5.88 (−7.23, −4.53) *	−5.91 (−7.49, −4.32) *	−6.26 (−7.77, −4.75) *	< 0.001
Psychosocial health summary score	Ref	−3.07 (−3.85, −2.30) *	−4.83 (−5.77, −3.88) *	−7.55 (−8.48, −6.61) *	< 0.001
Total scale score	Ref	−3.11 (−3.94, −2.28) *	−4.80 (−5.81, −3.79) *	−7.26 (−8.24, −6.28) *	< 0.001
Model 2					
Physical functioning	Ref	−2.13 (−4.01, −0.25) *	−3.65 (−5.78, −1.51) *	−4.64 (−6.74, −2.55) *	< 0.001
Emotional functioning	Ref	−0.14 (−1.10, 0.83)	−3.26 (−4.52, −2.01) *	−7.34 (−8.65, −6.03) *	< 0.001
Social functioning	Ref	−1.67 (−2.71, −0.64) *	−3.33 (−4.56, −2.09) *	−4.48 (−5.72, −3.25) *	< 0.001
School functioning	Ref	−4.33 (−5.68, −2.98) *	−4.10 (−5.67, −2.52) *	−3.92 (−5.46, −2.38) *	0.001
Psychosocial health summary score	Ref	−2.05 (−2.81, −1.28) *	−3.56 (−4.49, −2.63) *	−5.25 (−6.20, −4.30) *	< 0.001
Total scale score	Ref	−2.07 (−2.88, −1.25) *	−3.58 (−4.57, −2.59) *	−5.10 (−6.09, −4.11) *	< 0.001

Model 1 was a crude model. Model 2 adjusted for children’s age, sex, single child status, and primary caregivers, as well as maternal age, marital status, educational background, monthly per-capita income, level of family harmony, and negative emotional states

*ACEs* adverse childhood experiences, *HRQOL* health-related quality of life, *CI* confidence interval

^*^P-value < 0.05

The sex-specific impact of cumulative maternal ACEs on HRQOL are presented in Table 3. In general, boys with experience of one or more maternal ACEs had significantly lower HRQOL sub-scores and total scores, except for emotional functioning, which showed significantly reduced scores in boys of mothers with two or more ACEs. In contrast, compared to girls of mothers without report of any ACE experience, one or more maternal ACEs could impair the school functioning and psychosocial health summary scale of HRQOL in girls, ≥ 2 maternal ACEs were associated with significantly lower scores in emotional functioning and total scale, and only ≥ 3 maternal ACEs had significantly detrimental impact on physical functioning and social functioning in girls. We also observed significant dose-response associations of maternal cumulative ACE exposure with children’s HRQOL sub-scores and total scores in both boys and girls (*p*-values for trend < 0.05), except for the association between maternal ACEs and school functioning in girls, which only showed borderline significance of trend (*p*-value for trend = 0.051). Nevertheless, there was not a significant moderation effect of sex in aforementioned associations (*p*-values > 0.05 for all HRQOL sub-scores and total scores).

**Table 3:** Sex-specific association between the number of maternal ACEs and children’s HRQOL

	β (95% CI) by number of maternal ACEs				*P-*value for trend
	0	1	2	≥ 3	
Boys					
Physical functioning	Ref	−2.81 (−5.41, −0.22) *	−4.71 (−7.76, −1.66) *	−5.11 (−8.03, −2.20) *	< 0.001
Emotional functioning	Ref	−0.43 (−1.71, 0.85)	−3.04 (−4.73, −1.35) *	−6.78 (−8.53, −5.03) *	< 0.001
Social functioning	Ref	−2.56 (−3.96, −1.15) *	−4.11 (−5.83, −2.39) *	−5.54 (−7.28, −3.80) *	< 0.001
School functioning	Ref	−5.33 (−7.17, −3.49) *	−3.94 (−6.11, −1.76) *	−4.80 (−6.90, −2.69) *	0.011
Psychosocial health summary score	Ref	−2.77 (−3.81, −1.73) *	−3.70 (−4.99, −2.40) *	−5.70 (−7.02, −4.39) *	< 0.001
Total scale score	Ref	−2.78 (−3.90, −1.66) *	−3.95 (−5.34, −2.55) *	−5.56 (−6.93, −4.18) *	< 0.001
Girls					
Physical functioning	Ref	−1.40 (−4.13, 1.32)	−2.59 (−5.60, 0.41)	−4.11 (−7.14, −1.08) *	0.003
Emotional functioning	Ref	0.23 (−1.23, 1.70)	−3.47 (−5.35, −1.60) *	−7.88 (−9.86, −5.90) *	< 0.001
Social functioning	Ref	−0.79 (−2.32, 0.74)	−2.56 (−4.33, −0.78)	−3.38 (−5.13, −1.63) *	< 0.001
School functioning	Ref	−3.15 (−5.13, −1.16) *	−4.09 (−6.38, −1.80) *	−2.88 (−5.16, −0.60) *	0.051
Psychosocial health summary score	Ref	−1.24 (−2.37, −0.10) *	−3.37 (−4.72, −2.03) *	−4.72 (−6.10, −3.33) *	< 0.001
Total scale score	Ref	−1.28 (−2.47, 0.08)	−3.18 (−4.59, −1.77) *	−4.56 (−6.00, −3.13) *	< 0.001

Models adjusted for children’s age, single child status, and primary caregivers, as well as maternal age, marital status, educational background, monthly per-capita income, level of family harmony, and negative emotional states

*ACEs* adverse childhood experiences, *HRQOL* health-related quality of life, *CI* confidence interval

^*^P-value < 0.05

## Discussion

In this cross-sectional study, our results revealed that preschool children of mothers who were exposed to ACEs had worse HRQOL, compared to their counterparts of mothers who had no experience of any childhood adversity. Furthermore, the numbers of ACEs that mothers had experienced were associated with lower scores of children’s HRQOL in a dose-response pattern. Stratified analysis suggested that children’s sex did not moderate the associations between maternal ACEs and HRQOL in the offspring.

Findings regarding the negative associations between maternal ACEs and their children’s HRQOL were in line with several previous studies that have focused on other outcomes in children [11–16]. For example, a study of children aged between 0 and 17 years has shown that children of mothers with four or more ACEs had increased odds of hyperactivity (OR 3.10, 95% CI 1.5–6.2, *p*-value < 0.01) and emotional or mental disturbance (OR 5.66; 95% CI 2.0–15.9, *p*-value < 0.01), compared to children of mothers without any exposure to ACEs [16]. This was further demonstrated by a large cross-sectional study in China showing that maternal ACEs were significantly associated with more behavioral problems in their children aged between 3 and 6 years (OR 2.91, 95% CI 2.45–3.45, *p*-value < 0.001) [13]. Another cross-sectional survey has also proven that each additional parental ACE exposure was linked to 19% increased risk of poor overall health in their children (OR 1.19, 95% CI 1.07–1.32, *p*-value < 0.05) [14]. In addition, a retrospective cohort study has found that offspring of mothers with three or more ACEs had higher risk of suspected developmental delay, compared to their counterparts whose mothers were exposed to less than three types of childhood adversities [relative risk (RR) 2.23, 95% CI 1.37–3.63, *p*-value < 0.01) [11].

The exact mechanisms underlying the intergenerational association between ACEs and children’s HRQOL have not been fully elucidated. Previous research has shown that mothers who were exposed to ACEs tended to have negative health conditions (e.g., depression and obesity) and unhealthy lifestyles, including smoking and drug addiction [1, 38], which may be sustained throughout the period of pregnancy [39, 40]. According to the Developmental Origins of Health and Disease hypothesis, detrimental exposure during prenatal and perinatal periods could influence the health in later childhood and adult life [41]. Therefore, negative behaviors resulting in part from maternal ACEs may affect the normal development of fetus and lead to poorer HRQOL in their children through the unfavored gestational uterine environment. Also, the high stress originated from ACE exposure could increase the level of maternal cortisol, a stress biomarker that is the primary hormonal end-product of the hypothalamic–pituitary–adrenal (HPA) axis [42]. Cortisol could cross both the placental barrier and the blood brain barrier, and directly influence brain development of the fetus and eventually lead to childhood cognitive and mental disorders [43]. Therefore, it is biologically plausible that the adverse effects of maternal ACEs could be transmitted to children and cause poorer HRQOL. In addition, mothers with experiences of ACEs usually practiced negative parenting styles towards their children [24, 44], which could subsequently lead to more behavioral and health problems in children, as well as reduced scores of children’s HRQOL [25–27]. Furthermore, ACEs have been demonstrated to be linked to poorer educational attainment, financial hardship, and higher odds of depression in mothers [45–47], which were all associated with impaired HRQOL in their children [48, 49]. These possible mechanisms indicated opportunities and means that might stop the intergenerational impact of maternal ACEs on their offspring. From one hand, screening women with childhood adversities before and during pregnancy in obstetric and gynecological clinicals might be a feasible way to identify vulnerable women and promote preventive interventions [50]. From the other hand, it is of paramount importance to identify mothers with ACEs and provide them with proper interventions about psychosocial health and proper parenting skills, which might stop the detrimental cycles of ACEs [51]. In addition, child care workers, such as pediatrics, teachers, and social workers, should pay special attention to the wellbeing of children who had mothers with experience of ACEs.

We have further investigated the sex-specific associations of maternal ACEs with children’s HRQOL. The pattern of the associations indicated that boys were more sensitive to maternal ACEs than girls. It might due to the gender bias of mother report since the mother was the sole reporter in our study. However, most of the characteristics between boys and girls were comparable in our study, which might partially explain the non-significant moderation effect of children’s sex. The findings were in line with a cross-sectional study in China, which has shown that the association between maternal cumulative ACE scores and preschool children’s behavioral problems was comparable in boys and girls [13]. However, when the associations between type-specific ACE and children’s behavioral problems were further examined, results showed that girls of mothers with emotional abuse had increased risk of conduct problems compared to boys [13]. Likewise, the odds of anxiety were also higher among girls than boys of mothers who had experienced physical abuse or community violence [13]. In contrast, a study of 907 mother–child dyads in Canada has found that boys had greater vulnerability to develop behavioral problems in mothers with experience of ACEs [15]. One possible explanation of the inconsistency between our findings and aforementioned studies might be the different outcomes investigated. Unlike other studies that focused on behavioral problems, we specifically looked at HRQOL. Another cause of the inconsistency might be the different ACE indicators that were included in different studies. Furthermore, previous research has demonstrated that socio-cultural factors could influence the perceptions of ACEs [52]. Accordingly, mothers from different cultures might react to ACEs in a different manner, and subsequently lead to the inconsistent findings. Nevertheless, future studies are still needed to explore whether there are sex-specific associations between maternal ACEs and their children’s health outcomes, in order to facilitate targeted interventions.

Our study has included a large number of mother–child dyads with enough statistical power. We have measured HRQOL as the outcome to reflect children’s overall health and tested the moderation effect of sex. Moreover, sensitivity analysis was also conducted to avoid underreport of children’s HRQOL in mothers with negative emotional states [53]. However, some limitations should be noted as well. First, the study sample was recruited only from one megacity in China. The conclusion should be interpreted with caution when extrapolated to other populations. Second, although retrospective measurement of ACEs was subjective to recall bias, its reliability was supported by previous report [54]. Nevertheless, future studies should evaluate whether prospective and retrospective ACEs of mothers have different associations with their children’s health outcomes. Third, our study used mother report of children’s HRQOL, which might cause potential reporter bias. Although HRQOL should be captured by self-report, young children were unable to answer the questionnaire and proxy report was an unavoidable issue faced by researchers in child health [55, 56]. Fourth, as shown in the power analyses, our study might be over-powered and therefore was more prone to detect statistically significant differences [57]. Interpretations of our findings should be cautious. Last, although we have adjusted for several confounders in the multivariate analysis, some reported risk factors of HRQOL were not included due to data unavailability [49, 55, 56].

## Conclusions

In conclusion, this study indicated that maternal ACEs were associated with subsequent generation’s HRQOL. Significant dose-response pattern was also observed between the number of maternal ACEs and poorer children’ HRQOL sub-scores and total scores, without statistically significant sex differences. Our findings added fuel to the ongoing research about the intergenerational transmission of maternal ACEs to health problems in offspring. It also emphasized the importance of screening maternal ACEs, which might help identify high-risk children of poorer HRQOL with targeted interventions. Further randomized controlled trials on this topic are necessary. In addition, studies that explore the mechanisms of the intergenerational associations are also needed.

## Supplementary Information

**Additional file 1:** **Table S1. **The questionnaire items of maternal ACEs. **Table S2.** Comparison of characteristics of children and mothers by children’ sex. **Table S3.** Sensitivity analysis of the association between the number of maternal ACEs and children’s HRQOL in mothers without negative emotional states

## Data Availability

The datasets generated and/or analyzed during the current study are not publicly available due to privacy but are available from the corresponding author on reasonable request.

## Abbreviations

ACEs: adverse childhood experiences
HRQOL: health-related quality of life
ACE-IQ: Adverse Childhood Experiences-International Questionnaire
PedsQL 4.0: Pediatric Quality of Life Inventory version 4.0
FHS-5: Family Harmony Scale-5
DASS-21: Depression Anxiety Stress Scales-21
HPA: hypothalamic-pituitary-adrenal
OR: odds ratio
CI: confidence interval
RR: relative risk
SD: standard deviation

## References

[1] Felitti VJ, Anda RF, Nordenberg D, Williamson DF, Spitz AM, Edwards V (1998). Relationship of childhood abuse and household dysfunction to many of the leading causes of death in adults. The Adverse Childhood Experiences (ACE) Study. Am J Prev Med.

[2] Boullier M, Blair M (2018). Adverse childhood experiences. Paediatr Child Health.

[3] Jimenez ME, Wade R, Lin Y, Morrow LM, Reichman NE (2016). Adverse experiences in early childhood and kindergarten outcomes. Pediatrics.

[4] McKelvey LM, Edge NC, Mesman GR, Whiteside-Mansell L, Bradley RH (2018). Adverse experiences in infancy and toddlerhood: relations to adaptive behavior and academic status in middle childhood. Child Abuse Negl.

[5] McKelvey LM, Saccente JE, Swindle TM (2019). Adverse childhood experiences in infancy and toddlerhood predict obesity and health outcomes in middle childhood. Child Obes.

[6] Elsenburg LK, van Wijk KJE, Liefbroer AC, Smidt N (2017). Accumulation of adverse childhood events and overweight in children: a systematic review and meta-analysis. Obesity (Silver Spring).

[7] Lin L, Wang HH, Lu C, Chen W, Guo VY (2021). Adverse childhood experiences and subsequent chronic diseases among middle-aged or older adults in china and associations with demographic and socioeconomic characteristics. JAMA Netw Open.

[8] Lin L, Cao B, Chen W, Li J, Zhang Y, Guo VY (2022). Association of adverse childhood experiences and social isolation with later-life cognitive function among adults in China. JAMA Netw Open.

[9] Lin L, Sun W, Lu C, Chen W, Guo VY (2022). Adverse childhood experiences and handgrip strength among middle-aged and older adults: a cross-sectional study in China. BMC Geriatr.

[10] Hughes K, Bellis MA, Hardcastle KA, Sethi D, Butchart A, Mikton C (2017). The effect of multiple adverse childhood experiences on health: a systematic review and meta-analysis. Lancet Public Health.

[11] Folger AT, Eismann EA, Stephenson NB, Shapiro RA, Macaluso M, Brownrigg ME (2018). Parental adverse childhood experiences and offspring development at 2 years of age. Pediatrics.

[12] Cooke JE, Racine N, Pador P, Madigan S (2021). Maternal adverse childhood experiences and child behavior problems: a systematic review. Pediatrics.

[13] Wang X, Yin G, Guo F, Hu H, Jiang Z, Li S (2022). Associations of maternal adverse childhood experiences with behavioral problems in preschool children. J Interpers Violence.

[14] Lê-Scherban F, Wang X, Boyle-Steed KH, Pachter LM (2018). Intergenerational associations of parent adverse childhood experiences and child health outcomes. Pediatrics.

[15] Letourneau N, Dewey D, Kaplan BJ, Ntanda H, Novick J, Thomas JC (2019). Intergenerational transmission of adverse childhood experiences via maternal depression and anxiety and moderation by child sex. J Dev Orig Health Dis.

[16] Schickedanz A, Halfon N, Sastry N, Chung PJ (2018). Parents' adverse childhood experiences and their children's behavioral health problems. Pediatrics.

[17] Guyatt GH, Feeny DH, Patrick DL (1993). Measuring health-related quality of life. Ann Intern Med.

[18] Phyo AZZ, Gonzalez-Chica DA, Stocks NP, Storey E, Woods RL, Murray AM (2021). The utility of assessing health-related quality of life to predict cognitive decline and dementia. J Alzheimers Dis.

[19] Pinheiro LC, Reshetnyak E, Sterling MR, Richman JS, Kern LM, Safford MM (2019). Using health-related quality of life to predict cardiovascular disease events. Qual Life Res.

[20] Gobbens RJJ, van der Ploeg T (2021). The prediction of mortality by quality of life assessed with the WHOQOL-BREF: a longitudinal analysis at the domain and item levels using a seven-year follow-up period. Qual Life Res.

[21] Cohrdes C, Mauz E (2020). Self-efficacy and emotional stability buffer negative effects of adverse childhood experiences on young adult health-related quality of life. J Adolesc Health.

[22] Liming KW (2019). Examining the differing effects of economic hardship and poor maternal wellbeing on cumulative exposure to adverse childhood experiences. J Child Adolesc Trauma.

[23] Liming KW, Grube WAJC, Journal ASW (2018). Wellbeing outcomes for children exposed to multiple adverse experiences in early childhood: a systematic review. Child Adolesc Social Work J.

[24] Lange BCL, Callinan LS, Smith MV (2019). Adverse childhood experiences and their relation to parenting stress and parenting practices. Community Ment Health J.

[25] Guo Y, Zhang Y-Q, Wu C-A, Yin X-N, Zhang J-Y, Wu J-B (2022). Bidirectional associations between parenting styles and conduct problems in Chinese preschool children: the Shenzhen Longhua Child Cohort Study. Psychol Health Med.

[26] Byrne ML, Badcock PB, Simmons JG, Whittle S, Pettitt A, Olsson CA (2017). Self-reported parenting style is associated with children's inflammation and immune activation. J Fam Psychol.

[27] Sanavi FS, Baghbanian A, Shovey MF, Ansari-Moghaddam A (2013). A study on family communication pattern and parenting styles with quality of life in adolescent. J Pak Med Assoc.

[28] Luo S, Lin L, Chen W, Li C, Ren Y, Zhang M (2022). Association between maternal intimate partner violence and health-related quality of life in their preschool children: the mediating role of maternal parenting styles. Front Psychiatry.

[29] Wang BZK (2022). The differences between county, county-level city and municipal district in the system of administrative divisions in China. J Geogr Res.

[30] World Health Organization. Adverse Childhood Experiences International Questionnaire (ACE-IQ). https://www.who.int/publications/m/item/adverse-childhood-experiences-international-questionnaire-(ace-iq). Accessed 20 Sep 2022.

[31] Ho GWK, Chan ACY, Chien W-T, Bressington DT, Karatzias T (2019). Examining patterns of adversity in Chinese young adults using the Adverse Childhood Experiences-International Questionnaire (ACE-IQ). Child Abuse Negl.

[32] Varni JW, Seid M, Kurtin PS (2001). PedsQL 4.0: reliability and validity of the Pediatric Quality of Life Inventory version 4.0 generic core scales in healthy and patient populations. Med Care.

[33] Hao Y, Tian Q, Lu Y, Chai Y, Rao S (2010). Psychometric properties of the Chinese version of the Pediatric Quality of Life Inventory 4.0 generic core scales. Qual Life Res.

[34] Kavikondala S, Stewart SM, Ni MY, Chan BHY, Lee PH, Li K-K (2016). Structure and validity of Family Harmony Scale: an instrument for measuring harmony. Psychol Assess.

[35] Lovibond PF, Lovibond SH. The structure of negative emotional states: comparison of the depression anxiety stress scales (DASS) with the Beck depression and anxiety inventories. Behav Res Ther. 1995;33(3):335–43 10.1016/0005-7967(94)00075-u 7726811

[36] Wang K, Shi H-S, Geng F-L, Zou L-Q, Tan S-P, Wang Y (2016). Cross-cultural validation of the Depression Anxiety Stress Scale-21 in China. Psychol Assess.

[37] Richters J, Pellegrini D (1989). Depressed mothers' judgments about their children: an examination of the depression-distortion hypothesis. Child Dev.

[38] Afifi TO, Boman J, Fleisher W, Sareen J (2009). The relationship between child abuse, parental divorce, and lifetime mental disorders and suicidality in a nationally representative adult sample. Child Abuse Negl.

[39] Barrios YV, Gelaye B, Zhong Q, Nicolaidis C, Rondon MB, Garcia PJ (2015). Association of childhood physical and sexual abuse with intimate partner violence, poor general health and depressive symptoms among pregnant women. PLoS ONE.

[40] Nagl M, Steinig J, Klinitzke G, Stepan H, Kersting A (2016). Childhood maltreatment and pre-pregnancy obesity: a comparison of obese, overweight, and normal weight pregnant women. Arch Womens Ment Health.

[41] Gillman MW (2005). Developmental origins of health and disease. N Engl J Med.

[42] Thomas JC, Magel C, Tomfohr-Madsen L, Madigan S, Letourneau N, Campbell TS (2018). Adverse childhood experiences and HPA axis function in pregnant women. Horm Behav.

[43] Lupien SJ, McEwen BS, Gunnar MR, Heim C (2009). Effects of stress throughout the lifespan on the brain, behaviour and cognition. Nat Rev Neurosci.

[44] Wong RSM, Yu EYT, Guo VY, Wan EY-F, Chin W-Y, Wong CKH (2018). A prospective cohort study to investigate parental stress and child health in low-income Chinese families: protocol paper. BMJ Open.

[45] Houtepen LC, Heron J, Suderman MJ, Fraser A, Chittleborough CR, Howe LD (2020). Associations of adverse childhood experiences with educational attainment and adolescent health and the role of family and socioeconomic factors: a prospective cohort study in the UK. PLoS Med.

[46] Racine N, Devereaux C, Cooke JE, Eirich R, Zhu J, Madigan S (2021). Adverse childhood experiences and maternal anxiety and depression: a meta-analysis. BMC Psychiatry.

[47] Lin L, Cao B, Chen W, Li J, Zhang Y, Guo VY (2022). Association of childhood threat and deprivation with depressive symptoms and the moderating role of current economic status among middle-aged and older adults in China. Soc Psychiatry Psychiatr Epidemiol.

[48] Von Rueden U, Gosch A, Rajmil L, Bisegger C, Ravens-Sieberer U (2006). Socioeconomic determinants of health related quality of life in childhood and adolescence: results from a European study. J Epidemiol Community Health.

[49] Phua DY, Kee MZL, Meaney MJ (2020). Positive Maternal mental health, parenting, and child development. Biol Psychiatry.

[50] Rariden C, SmithBattle L, Yoo JH, Cibulka N, Loman D (2021). Screening for adverse childhood experiences: literature review and practice implications. Journal Nurse Pract.

[51] Muzik M, Rosenblum KL, Alfafara EA, Schuster MM, Miller NM, Waddell RM (2015). Mom power: preliminary outcomes of a group intervention to improve mental health and parenting among high-risk mothers. Arch Womens Ment Health.

[52] Grest CV, Finno-Velasquez M, Cederbaum JA, Unger JB (2021). Adverse childhood experiences among 3 generations of Latinx youth. Am J Prev Med.

[53] Chilcoat HD, Breslau N (1997). Does psychiatric history bias mothers' reports? An application of a new analytic approach. J Am Acad Child Adolesc Psychiatry.

[54] Dube SR, Williamson DF, Thompson T, Felitti VJ, Anda RF (2004). Assessing the reliability of retrospective reports of adverse childhood experiences among adult HMO members attending a primary care clinic. Child Abuse Negl.

[55] Guo VY, Yu EYT, Wong RSM, Ip P, Tiwari AFY, Wong CKH (2017). Maternal mental quality of life mediates the associations between intimate partner abuse against mothers and their children's behaviours and quality of life in low-income Chinese families. Qual Life Res.

[56] Guo VY, Wong CKH, Wong RSM, Yu EYT, Ip P, Lam CLK (2018). Spillover effects of maternal chronic disease on children's quality of life and behaviors among low-income families. Patient.

[57] Sullivan GM, RJJogme F (2012). Using effect size—or why the P value is not enough. J Grad Med Educ.

