# Supplementary material for: Maternal adverse childhood experiences and health-related quality of life in preschool children: a cross-sectional study
Source: Child Adolesc Psychiatry Ment Health. 2023 Feb 6;17:19. doi: 10.1186/s13034-023-00570-6 (PMC9903527; doi:10.1186/s13034-023-00570-6)
Supplement: Supplementary file 1 — Additional file 1: Table S1. The questionnaire items of maternal ACEs. Table S2. Comparison of characteristics of children and mothers by children’ sex. Table S3. Sensitivity analysis of the association between the number of maternal ACEs and children’s HRQOL in mothers without negative emotional states [file 13034_2023_570_MOESM1_ESM.docx]

***Child and Adolescent Psychiatry and Mental Health***

Maternal adverse childhood experiences and health-related quality of life in preschool children: a cross-sectional study

**Author:** Dezhong Chen BA ^a^, Li Lin BA ^a^, Chunrong Li PhD ^b^, Weiqing Chen PhD ^a^, Yuying Zhang PhD, MD ^c^, Yan Ren MS ^b^, Vivian Yawei Guo PhD ^a^

**Affiliation:**

^a^ Department of Epidemiology, School of Public Health, Sun Yat-sen University, Guangzhou, China;

^b^ Chengdu Women’s and Children’s Central Hospital, School of Medicine, University of Electronic Science and Technology of China, Chengdu, China;

^c^ Department of Child Healthcare, Shenzhen Longhua Maternity and Child Healthcare Hospital, Shenzhen, China

**Address correspondence to:** Vivian Yawei Guo, Ph.D., Department of Epidemiology, School of Public Health, Sun Yat-sen University, 74 Zhongshan Second Road, Guangzhou, Guangdong 510080, China.

Email: [guoyw23@mail.sysu.edu.cn](mailto:guoyw23@mail.sysu.edu.cn)

**Table S1. The questionnaire items of maternal ACEs**

| All questions were preceded by the statement “While you were growing up, before the age of 18 …” | | | |
| --- | --- | --- | --- |
| ACE indicator | Questionnaire Items | N | Prevalence (%) |
| Physical abuse | Did a parent, guardian or other household member spank, slap, kick, punch or beat you up? (Never, once*, a few times*, or many times*) | 618 | 14.6% |
|  | Did a parent, guardian or other household member hit or cut you with an object, such as a stick (or cane), bottle, club, knife, whip etc.? (Never, once*, a few times*, or many times*) |  |  |
| Emotional abuse | Did a parent, guardian or other household member yell, scream or swear at you, insult or humiliate you? (Never, once*, a few times*, or many times*) | 657 | 15.5% |
|  | Did a parent, guardian or other household member threaten to, or actually, abandon you or throw you out of the house? (Never, once*, a few times*, or many times*) |  |  |
| Physical neglect | How often did your parents/guardians not give you enough food even when they could easily have done so? (Never, once*, a few times*, or many times*) | 95 | 2.2% |
|  | Were your parents/guardians too drunk or intoxicated by drugs to take care of you? (Never, once*, a few times*, or many times*) |  |  |
|  | How often did your parents/guardians not send you to school even when it was available? (Never, once*, a few times*, or many times*) |  |  |
| Emotional neglect | Did your parents/guardians understand your problems and worries? (Never*, rarely*, sometimes*, most of time, or always) | 3,312 | 78.1% |
|  | Did your parents/guardians really know what you were doing with your free time when you were not at school or work? (Never*, rarely*, sometimes*, most of time, or always) |  |  |
| Household substance abuse | Did you live with a household member who was a problem drinker or alcoholic, or misused street or prescription drugs? (Yes* or no) | 9 | 0.2% |
| Incarcerated household member | Did you live with a household member who was ever sent to jail or prison? (Yes* or no) | 9 | 0.2% |
| Mental illness in household | Did you live with a household member who was depressed, mentally ill or suicidal? (Yes* or no) | 14 | 0.3% |
| Intimate partner violence | Did you see or hear a parent or household member in your home being yelled at, screamed at, sworn at, insulted or humiliated? (Never, once*, a few times*, or many times*) | 600 | 14.1% |
|  | Did you see or hear a parent or household member in your home being slapped, kicked, punched or beaten up? (Never, once*, a few times*, or many times*) |  |  |
|  | Did you see or hear a parent or household member in your home being hit or cut with an object, such as a stick (or cane), bottle, club, knife, whip etc.? (Never, once*, a few times*, or many times*) |  |  |
| Parental separation or divorce | Were your parents ever separated or divorced? (Yes* or no) | 307 | 7.2% |
| Parental death | Did your mother, father or guardian die? (Yes* or no) | 186 | 4.4% |
| Bully | How often were you bullied? (Never, once*, a few times*, or many times*) | 320 | 7.5% |
| Community violence | Did you see or hear someone being beaten up in real life? (Never, once*, a few times*, or many times*) | 1,270 | 29.9% |
|  | Did you see or hear someone being stabbed or shot in real life? (Never, once*, a few times*, or many times*) |  |  |
|  | Did you see or hear someone being threatened with a knife or gun in real life? (Never, once*, a few times*, or many times*) |  |  |

* Indicate answers thresholds for ACEs

ACEs: adverse childhood experiences.

**Table S2. Comparison of characteristics of children and mothers by children’ sex**

| **Characteristics** | | | | **Sex** | | ***P*-value for difference** |
| --- | --- | --- | --- | --- | --- | --- |
|  |  |  |  | **Boys (n = 2,193)** | **Girls (n= 2,050)** |  |
| **Maternal characteristics** | | | |  |  |  |
|  | Age (years), mean ± SD | | | 33.1 ± 4.6 | 33.1 ± 4.6 | 0.859 |
|  | Marital status, n (%) | | |  |  |  |
|  |  | | Married | 2,123 (96.8%) | 1,967 (96.0%) |  |
|  |  | | Unmarried | 70 (3.2%) | 83 (4.0%) |  |
|  | Educational background, n (%) | | |  |  | 0.188 |
|  |  | | Junior high school or below | 217 (9.9%) | 170 (8.3%) |  |
|  |  | | Senior high school | 503 (23.0%) | 485 (23.7%) |  |
|  |  | | Bachelor degree or above | 1,471 (67.1%) | 1,393 (68.0%) |  |
|  | Monthly per-capita income, n (%) | | |  |  | 0.357 |
|  |  | | ≤ 5,000 RMB | 598 (27.3%) | 577 (28.1%) |  |
|  |  | | 5,001-10,000 RMB | 596 (27.2%) | 510 (24.9%) |  |
|  |  | | > 10,000 RMB | 722 (32.9%) | 707 (34.5%) |  |
|  |  | | Uncertain | 277 (12.6%) | 256 (12.5%) |  |
|  | Negative emotional states, n (%) | | |  |  |  |
|  |  | | Depression | 97 (4.4%) | 121 (5.9%) | 0.029^*^ |
|  |  | | Anxiety | 145 (6.6%) | 149 (7.3%) | 0.400 |
|  |  | | Stress | 125 (5.7%) | 119 (5.8%) | 0.883 |
|  | Level of family harmony, mean ± SD | | | 21.0 ± 4.6 | 20.9 ± 4.6 | 0.583 |
|  | ACEs, n (%) | | |  |  | 0.339 |
|  |  | 0 | | 320 (14.6%) | 282 (13.8%) |  |
|  |  | 1 | | 1,012 (46.1%) | 907 (44.2%) |  |
|  |  | 2 | | 385 (17.6%) | 389 (19.0%) |  |
|  |  | ≥ 3 | | 476 (21.7%) | 472 (23.0%) |  |
| **Child characteristics** | | | |  |  |  |
|  | Age (years), mean ± SD | | | 4.6 ± 1.0 | 4.6 ± 1.0 | 0.732 |
|  | Status of single child, n (%) | | |  |  | 0.601 |
|  |  | Yes | | 1,003 (45.7%) | 954 (46.5%) |  |
|  |  | No | | 1,190 (54.3%) | 1,096 (53.5%) |  |
|  | Primary caregivers, n (%) | | |  |  | 0.071 |
|  |  | | Mothers | 1,621 (73.9%) | 1,516 (74.0%) |  |
|  |  | | Fathers | 89 (4.1%) | 58 (2.8%) |  |
|  |  | | Grandparents or other people | 483 (22.0%) | 476 (23.2%) |  |
|  | HRQOL, mean ± SD | | |  |  |  |
|  |  | | Physical functioning | 81.7 ± 20.7 | 82.6 ± 19.9 | 0.143 |
|  |  | | Emotional functioning | 91.4 ± 12.9 | 90.9 ± 13.7 | 0.297 |
|  |  | | Social functioning | 76.9 ± 11.8 | 76.8 ± 11.6 | 0.730 |
|  |  | | School functioning | 70.0 ± 15.0 | 71.1 ± 15.2 | 0.015^*^ |
|  |  | | Psychosocial health summary score | 79.4 ± 9.3 | 79.6 ± 9.4 | 0.498 |
|  |  | | Total scale score | 80.0 ± 9.9 | 80.4 ± 9.8 | 0.214 |

* *P*-value < 0.05

Abbreviations: ACEs adverse childhood experiences, HRQOL: health-related quality of life, SD: standard deviation.

**Table S3. Sensitivity analysis** **of the association between the number of maternal ACEs and children’s HRQOL in mothers without negative emotional states**

|  |  | **β (95% CI) by number of maternal ACEs** | | | | ***P*-value for trend** |
| --- | --- | --- | --- | --- | --- | --- |
|  |  | **0** | **1** | **2** | **≥ 3** |  |
|  | Physical functioning | Ref | -2.41 (-4.32, -0.51) * | -3.31 (-5.50, -1.13) * | -4.87 (-7.05, -2.68) * | < 0.001 |
|  | Emotional functioning | Ref | 0.03 (-0.93, 0.98) | -3.12 (-4.39, -1.85) * | -7.64 (-8.99, -6.29) * | < 0.001 |
|  | Social functioning | Ref | -1.77 (-2.81, -0.72) * | -3.31 (-4.57, -2.04) * | -4.73 (-6.01, -3.44) * | < 0.001 |
|  | School functioning | Ref | -4.29 (-5.66, -2.91) * | -4.13 (-5.75, -2.51) * | -4.23 (-5.84, -2.61) * | < 0.001 |
|  | Psychosocial health summary score | Ref | -2.01 (-2.78, -1.24) * | -3.52 (-4.47, -2.57) * | -5.53 (-6.52, -4.55) * | < 0.001 |
|  | Total scale score | Ref | -2.21 (-3.31, -1.11) * | -3.42 (-4.73, -2.10) * | -5.20 (-6.51, -3.89) * | < 0.001 |

* *P*-value < 0.05.

ACEs, adverse childhood experiences; CI, confidence interval; HRQOL: health-related quality of life. Models adjusted for children’s age, sex, single child status, and primary caregivers, as well as maternal age, marital status, educational background, monthly per-capita income, and level of family harmony.
